# Easing the strain of fetal cardiovascular magnetic resonance: Editorial for “Fetal cardiovascular magnetic resonance feature tracking myocardial strain analysis in congenital heart disease”

**DOI:** 10.1016/j.jocmr.2024.101115

**Published:** 2024-10-28

**Authors:** Alex J. Barker, Lorna P. Browne, Richard M. Friesen

**Affiliations:** Department of Radiology, Section of Pediatric Radiology, Children’s Hospital Colorado, University of Colorado Anschutz Medical Campus, Aurora, Colorado, USA; Department of Bioengineering, University of Colorado Anschutz Medical Campus, Aurora, Colorado, USA; Department of Radiology, Section of Pediatric Radiology, Children’s Hospital Colorado, University of Colorado Anschutz Medical Campus, Aurora, Colorado, USA; Department of Pediatrics, Section of Pediatric Cardiology, Children’s Hospital Colorado, University of Colorado, Anschutz Medical Campus, Aurora, Colorado, USA

The reality of performing fetal cardiovascular magnetic resonance (CMR) in a busy clinical practice, with timely on-scanner image reconstruction, and accompanying commercially available software to interpret images, would have been considered a fantasy a few short years ago. In addition to the technical difficulty associated with a lack of an electrocardiogram signal for cardiac gating, fetal CMR brings its own set of intrinsic challenges, including high heart rates (120–160 bpm), small cardiovascular structures, a lack of ability to use exogenous contrast agents, and a high probability that the baby will perform mid-acquisition gymnastics. Fortunately, the recent emergence of a United States Food and Drug Administration- and Conformité Européenne-approved cardiotocographic device capable of synchronizing scanner data acquisition with the fetal cardiac cycle has eased the strain caused by one such obstacle to clinical translation [1]. Before this, fetal CMR was not widely available beyond expert centers with custom, offline reconstruction workflows [2], [3]. With the emergence of regulatory approval for the device, the ability to broadly use this technique in third-trimester babies (larger fetal size limits some gross movement) has facilitated recent publications providing insight into CMR-derived cardiac function in the setting of healthy physiology and congenital heart disease (CHD) [4], [5].

These recent insights are further augmented by Vollbrecht et al. in their recent paper, “*Fetal Cardiovascular Magnetic Resonance Feature Tracking Myocardial Strain Analysis in Congenital Heart Disease”* [6], where remarkable success is reported in acquiring dynamic cine balanced steady-state free precession images of axial and four-chamber views in 60 third-trimester fetuses. In this retrospective analysis of fetuses suspected of cardiovascular disease or thoracic malformations, Vollbrecht et al. were able to perform fetal myocardial deformation analysis using CMR-based feature tracking with an optimized version of commercially available software. Postnatal echocardiography and/or surgical reports were used to confirm prenatal diagnosis. In their cohort, a broad spectrum of healthy and CHDs cases were recorded, including 8 healthy controls and a distribution of patients with hypoplastic left heart syndrome (HLHS, N = 13), d-transposition of the great arteries (N = 10), coarctation of the aorta (CoA, N = 9), tetralogy of Fallot (N = 7), atrioventricular septal defects (AVSD, N = 7), and pulmonary valve stenosis/atresia (N = 6).

In addition to the 60 successfully acquired exams, there were two exams with inadequate image quality, an acquisition failure rate of 3% (2/60), which is commendable given the known challenges associated with fetal CMR. Furthermore, they performed feature tracking strain analysis of a four-chamber view of the left- and right ventricles (LV, RV) to obtain LV global longitudinal strain (GLS) and strain rate, as well as LV global radial strain and RV GLS. It again is encouraging that they yielded a software postprocessing success rate of 113 out of 120 ventricles (94%). Given the challenges with motion during acquisition, small sizes, and limited spatial resolution, it is reassuring that the images yielded enough quality to allow for myocardial feature tracking. Furthermore, the intra- and inter-observer intraclass correlation coefficients (ICCs) were excellent (ICC > 0.9) with only the RV GLS being rated as good (ICC < 0.9). Finally, of the seven failed ventricles, six were caused by the ventricles being too small in HLHS and AVSD patients, an understandable reason for the failure. The success rates and inter- and intra-observer repeatability are commendable and lend confidence to the use of fetal CMR functional analysis for prenatal evaluation of CHD.

Yet, even in the presence of these encouraging results, it must be acknowledged that evaluation of ventricular strain by CMR feature tracking is an imperfect tool subject to spatial and temporal resolution dependencies. Taking this into consideration, ventricular strain has still been shown to be of value across a spectrum of congenital and acquired forms of pediatric cardiac disease [7], [8]. Thus, with recognition that some of the spatiotemporal dependencies are likely heightened in the fetus, the authors’ assessment of ventricular strain is an exciting proof of concept, highlighting the potential of the technique to understand fetal cardiac physiology in the presence of CHD. In their article, ventricular strain was calculated across various CHD conditions with encouraging success. Although it is expected that LV GLS is significantly decreased in HLHS and right dominant AVSD lesions, the ability to demonstrate this with fetal CMR presents an interesting possibility of the role of fetal strain in cases of borderline left ventricular sizes and for predicting the success of a biventricular repair.

Furthermore, the challenge of prenatal diagnosis of CoA is well known, with fetal CMR showing great promise for improving prenatal diagnosis using flow and morphology [9], but literature on ventricular function by fetal CMR is lacking. Ventricular strain measurements in patients with CoA may represent an additional biomarker of disease; however, care must be taken, especially when considering the possibility of aortic valve disease. This is a potential confounder both in this study and in the prior echocardiographic studies and needs to be further clarified in larger cohorts. The studies referencing an increased LV GLS mentioned in this paper also note the presence of aortic stenosis along with coarctation, which represents a different prenatal physiology than isolated CoA and which may explain some of the presumed adaptation of the ventricular strain in the presence of increased afterload. A fetal echocardiographic study of CoA *without* aortic stenosis demonstrated *decreased* LV GLS by fetal echocardiography, rather than *increased*, with no difference in RV GLS, which contrasts with the CMR findings here [10]. While the clinical implementation of the current findings must be considered in the context of cohort heterogeneity, the observation of biventricular strain differences by fetal CMR across varieties of CHD offers an opportunity to further our understanding prenatal physiology and adaptation in CHD.

Some missing pieces to the fetal CHD puzzle that are not presented here are ventricular size data and standard functional measurements (such as ejection fraction or shortening fraction). These data require a stack of cine images in the short-axis plane and will result in longer exam times and more potential for fetal motion and failed acquisitions. Vollbrecht et al. do not report acquiring these images. This may have resulted in the high rate of successful strain analyses due to the limited views acquired, but an idealized approach would be to attempt a comprehensive analysis of function and size to increase our understanding of the fetal physiology in the presence of disease.

It is exciting that the evaluation of ventricular strain was shown here to be feasible in a vast majority of patients. For centers interested in starting up a fetal CMR service, they should be careful to consider that there is a learning curve before overall success rates (including acquisition and postprocessing) of 94% can be achieved. This will take time and experience. Finally, we should remember that, until recently, CMR was not widely available for fetal myocardial deformation analysis, except in expert centers with custom, offline reconstruction workflows. Vollbrecht et al. are commended for demonstrating that the translation of this technology is clinically viable, in one of the larger functional fetal CMR cohorts reported to date, and for showing that one can apply the technology in a broad range of CHD patients with the expectation of a successful result. We look forward to future studies shedding additional light on their initial findings.

## Author contributions

**Alex J. Barker**: Writing—original draft, Writing – review & Writing—review and editing. **Lorna P. Browne**: Writing—original draft, Writing—review and editing. **Richard M. Friesen**: Writing—original draft, Writing—review and editing.

## Declaration of competing interests

The authors declare that they have no known competing financial interests or personal relationships that could have appeared to influence the work reported in this paper.
